# Supplementary material for: NRXN1 as a Prognostic Biomarker: Linking Copy Number Variation to EMT and Survival in Colon Cancer
Source: Int J Mol Sci. 2024 Oct 24;25(21):11423. doi: 10.3390/ijms252111423 (PMC11546699; doi:10.3390/ijms252111423)
Supplement: Supplementary file 1 [file ijms-25-11423-s001.zip › ijms-3196883-supplementary.pdf]

Supplementary Materials

Supplementary Figures

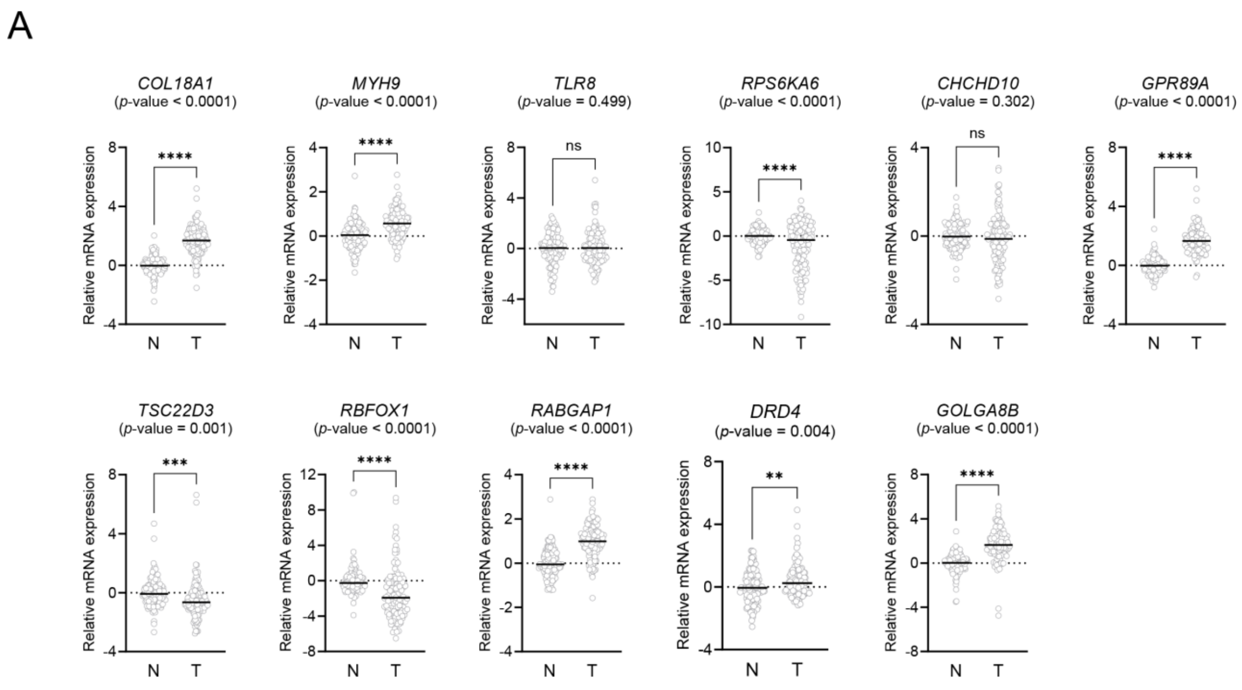

Figure S1. Gene expression analysis. Extended data related to Figure 1.

Differential gene expression of putative survival-related genes in tumor and paired normal tissues (stage II, III, n=120). N, normal tissues; T, tumor tissues. Data are presented as mean ± SD. ns, not significant; **p < 0.01; ***p < 0.001; ****p < 0.0001.

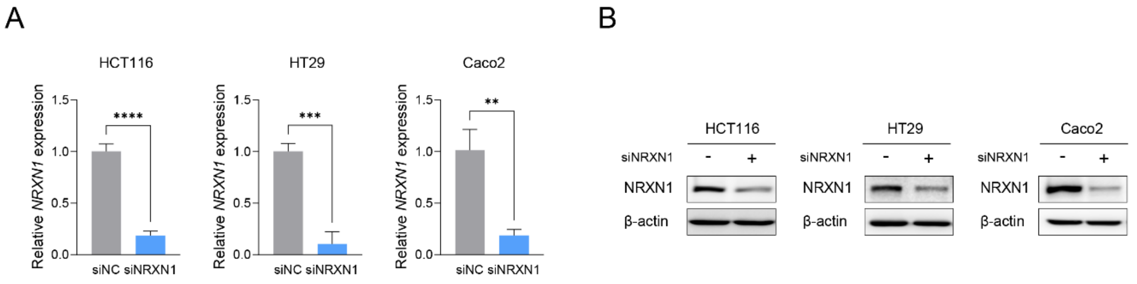

Figure S2. Validation of NRXN1 knockdown in siNRXN1-transfected cells. (A) RT-qPCR analysis confirmed the knockdown of NRXN1 mRNA levels in cells transfected with siNRXN1 compared to control cells. GAPDH was used as the control for normalization. Data are presented as mean ± SD. ns, not significant; **p < 0.01; ***p < 0.001; ****p < 0.0001.

(B) Western blot analysis verifying NRXN1 protein knockdown in siNRXN1-transfected cells. β-actin was used as the loading control.

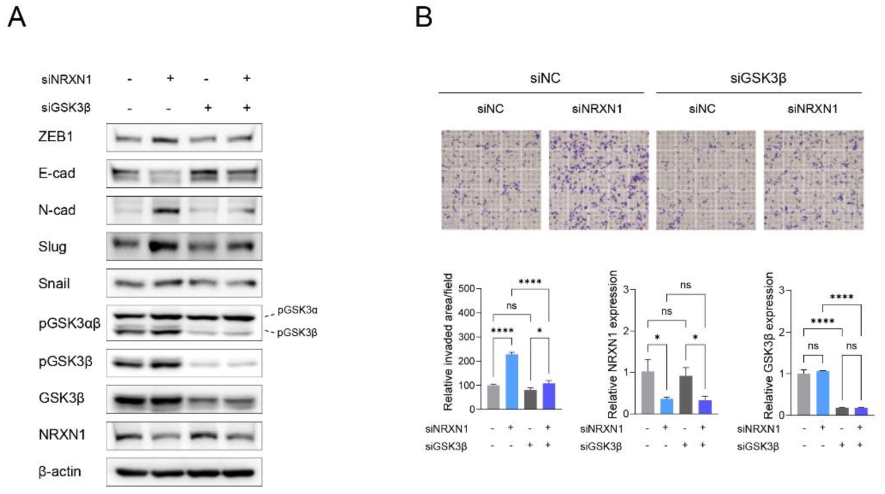

Figure S3. The effect of GSK3β and NRXN1 knockdown on cell invasion and EMT in HCT116 cells. (A) HCT116 cells were transfected with siNC (negative control), siNRXN1, siGSK3β, or both siNRXN1 and siGSK3β. Western blot analysis was performed 48 hours post-transfection to confirm the knockdown of genes and their effect on EMT signaling pathways. (B) Transwell invasion assays were conducted to evaluate the invasive properties of HCT16 cells upon NRXN1 and GSK3β knockdown. Representative images from the invasion assay and quantification of invasive cell counts are presented. RT-qPCR validated the successful knockdown of NRXN1 and GSK3β. The results indicate that NRXN1 knockdown promotes CRC cell invasion and EMT, while GSK3β knockdown inhibits NRXN1 knockdown-induced invasion and EMT. Data are presented as mean ± SD. ns; no significant, * $p < 0.05$, **** $p < 0.0001$.

Supplementary Tables

Table S1: Primers used for qRT-PCR.

Gene	Primer sequence

	Forward (5′-3′)	Reverse (5′-3′)
NRXN1	GCTATCTTGGCAGGTCCTGTGA	ACATCCTCAGCCTCCGTATGCA
ZEB1	GATGATGAATGCGAGTCAGATGC	ACAGCAGTGTCTTGTTGTTGT
E-cadherin	CGAGAGCTACACGTTCACGG	GGGTGTCGAGGGAAAAATAGG
N-cadherin	TGTTTGACTATGAAGGCAGTGG	TCAGTCATCACCTCCACCAT
Vimentin	GACGCCATCAACACCGAGTT	CTTTGTCGTTGGTTAGCTGGT
Snail	TCGGAAGCCTAACTACAGCGA	AGATGAGCATTGGCAGCGAG
Slug	CGAACTGGACACACATACAGTG	CTGAGGATCTCTGGTTGTGGT
Twist	GTCCGCAGTCTTACGAGGAG	GCTTGAGGGTCTGAATCTTGCT
GSK3β	AAGGATTCGTCAGGAACAGGA	ATGAATGTGCACAAGCTTCCA
GAPDH	GTGAAGGTCGGAGTCAAC	GTTGAGGTCAATGAAGGG

Table S2: Primary antibodies used in the study (Western blot analysis)

Antibody	Manufacturer	Cat. No.	Predicted size	Dilution
NRXN1	Novus	NBP 2-94557	160 kDa	1:1000
ZEB1	CST	70512	200 kDa	1:1000
E-cadherin	BD	610182	120 kDa	1:5000
N-cadherin	Abcam	ab18203	125-135 kDa	1:1000
Vimentin	CST	5741	57 kDa	1:1000
Snail	CST	3879	29 kDa	1:1000
Slug	CST	9585	30 kDa	1:1000
pGSK3αβ (S21/9)	CST	8566	α 51/β 46 kDa	1:1000
pGSK3β (S9)	CST	5558	46 kDa	1:1000
GSK3β	CST	9315	46 kDa	1:1000
β-actin	Abcam	ab8227	43 kDa	1:1000

β-actin	Abcam	ab6276	43 kDa	1:1000

All secondary HRP-conjugated antibodies for western blotting analysis were purchased from Jackson ImmunoResearch Laboratories. Secondary HRP-conjugated antibodies produced by Rockland were used for western blotting analysis of immunoprecipitated proteins.
